# Error in Figure

**DOI:** 10.1001/jamanetworkopen.2026.19326

**Published:** 2026-05-28

In the Original Investigation titled “Hypertrophic Cardiomyopathy and Risk of Out-of-Hospital Cardiac Arrest,” published April 29, 2026, the lines in the graph in Figure 2 were labeled incorrectly. This article has been corrected.^1^
